# Moving Beyond Simple Risk Prediction: Segmenting Patient Populations Using Consumer Data

**DOI:** 10.3389/fpubh.2021.716754

**Published:** 2021-07-15

**Authors:** Mandana Rezaeiahari

**Affiliations:** Department of Health Policy and Management, University of Arkansas for Medical Sciences, Little Rock, AR, United States

**Keywords:** consumer marketing, patient segmentation, population health, social determinants of health, risk stratification

## Introduction

There has been growing interest among health systems in population health (1, 2). Population health aims to improve the overall health of a population across the full continuum of care by more targeted, effective and coordinated health services (3). Given the rising trend of aging population and chronic disease burden, managing population health becomes more important for health systems trying to control cost (4).

In order to improve outcomes and efficiency, health systems need to customize care and interventions based on identified risks and costs (5). One of the systematic approaches in the literature for targeting interventions to subgroups of patients with different needs is population segmentation or risk-stratification (referred as *patient segmentation* in the remainder of this paper). Population segmentation that divides a population into groups with related service needs is an important foundation for effective and sustainable care delivery (6–8). Segmentation divides patients into distinct groups with specific needs, characteristics or behaviors and allows for health services to be organized around patients with similar needs (7). Patient segmentation models are becoming essential element of healthcare management due to the increase in the number of programs that incentivize value-based care (9).

Although patient segmentation models can help design interventions targeting subgroups of patients, they are often based on International Classification of Diseases (ICD) codes found in electronic health records (EHRs) and/or insurance claims data and lack important social risk factors that are essential for designing interventions. World Health Organization defines social determinants of health (SDOH) as the conditions in which people are born, grow, work, live, and age (10). These factors include economic policies and systems, development agendas, social norms, social policies and political systems (10). There are numerous studies demonstrating social factors acting as powerful determinants on multiple health outcomes including coronary heart disease (11), breast cancer (12), childhood obesity (13) and end-stage renal failure (14). Literature suggests that high utilizers of healthcare resources among Medicaid and uninsured population often have multiple chronic conditions (15, 16) and programs targeting this population collectively argue that social risk factors including but not limited to language, health literacy, unemployment, substance abuse and housing are important drivers of healthcare utilization (17, 18).

Most of the current patient segmentation models use administrative billing data because insurance claims data provides a nearly complete view of patients' interactions with health care delivery system; therefore, it is a reliable source to extract utilization outcomes (19). Majority of the EHRs on the other hand contain data from clinical encounters occurring between individuals and providers within a single health system and hence miss out of network events (19). On the positive side of EHRs is that they offer more extensive data including family history, lab results, vital signs and symptoms which could help improve the population segmentation model (20). One drawback of reliance on insurance claims data and EHRs is that they miss social and behavioral factors that complicate care (21). Although, there is a subset of ICD-10-CM codes, the Z codes, for documenting SDOH in EHRs, these codes are underutilized (22, 23). As such, SDOH Z codes may not reflect the actual burden of social needs experienced by patients. To address this gap, this paper presents the complementary benefit of consumer data when it is linked to EHRs or insurance claims data. The consumer marketing data include individual-level SDOH (including income, education, lifestyle variables, language spoken, household size, smoking status, life events, shopping activity) that are not available in the insurance claims data or majority of EHR data. The combined data provides 360-degree view of patients and can help predict the risk of repeat emergency room visits or hospital admissions (24). Inclusion of SDOH is essential to improve population health as medical interventions without addressing social determinants are not sustainable and effective. This unprecedented view into the lives of patients has significant potential to improve upon segmentation approaches relying exclusively on health plan or EHR data that lack measures or even decent proxies for fitness, diet and other SDOH which can profoundly alter the course of chronic diseases. A number of commercial companies provide marketing data that is well-utilized by organizations that subscribe to their services. Experian's ConsumerView^SM^ U.S. database is one of the world's largest consumer database on more than 300 million individuals and 126 million households (25). ConsumerView^SM^ U.S. database is compiled from hundreds of resources. For example, property and mortgage data are compiled from public records and county deeds while lifestyle and interest data are compiled from consumers who have completed self-reported surveys (25). Marketing companies match and mange patient identity across the healthcare ecosystems enabling the linkage of datasets across channels and silos (26, 27). According to Acxiom, two-thirds of hospitals actively use or want third-party consumer and lifestyle data to improve patient care (24).

## Current Patient Segmentation Models

There are two major approaches for conducting population segmentation in the literature. Expert-driven approaches are informed by expert consensus while data-driven approaches use statistical analysis such as clustering to segment a population (28). John Hopkins Adjusted Clinical Group (ACG) system and the Clinical Risk Group (CRG) system by 3M Health Information Systems are examples of expert-driven approaches (29, 30). The ACG system assigns each diagnosis code to one or more of 32 diagnosis groups referred to as Aggregated Diagnosis Groups (ADG). Both ACG and CRG system use diagnostic codes to classify patients into over 200 mutually exclusive risk groups (28). ADGs are assigned based on five features of conditions: duration, severity, diagnostic certainty, type of etiology and expected need for specialty care. The 3M CRG system assigns an individual five-digit classification code with first digit representing the core health status group, second through the fourth digit representing the base 3M CRG and the fifth digit identifying the severity-of-illness level (30). One drawback of expert-driven approaches is that they subjectively segment populations and no specific standards are set to derive the number of segments. Data-driven approaches generate evidence-based insights of population health status based on patient healthcare data to support policy decisions (1). There have been multiple studies using data-driven approaches to segment populations (19, 31, 32). Zhang et al. (33) developed a patient taxonomy with ten categories to divide high-cost Medicare Fee-For-Service patients. They found high-cost patients were most likely to have multiple chronic conditions, serious mental illness, serious medical illness and frailty (33). Low et al. (28) used cluster analysis and healthcare utilization data from electronic medical records to develop five segments of population (28). Concurrent with patient segmentation models developed by researchers, many predictive models based on SDOH have been developed by health payers and analytics companies. Most often these models are proprietary hence not available for review and scrutiny (34). For instance, a non-profit health insurance company used consumer data to develop a segmentation model to make informed adjustments to its Medicare marketing efforts (35).

## Discussion

As medical care is only responsible for 15 to 20% of preventable mortality in the US (36) and due to the increasing impact of social factors on health, it is now time to leverage data analytics to start to understand SDOH and its impact on health and design more social centered care coordination interventions (37). A recent critical review of patient segmentation models shows a lack of comprehensive models that integrates data from multiple sources, with a majority of the models limited to administrative billing data alone (21).

Healthcare organizations and payers should strive to link their traditional resources including EHRs and insurance claims data to consumer marketing data. Through this linkage, they can then apply advanced analytics to get tangible results that can be acted upon to improve quality of care and health outcomes. Specifically, more data driven approaches are needed to utilize available data to assess whether distinct patient subgroups might exist within population. For example, cluster analysis may be used to determine if individual level SDOH (based on consumer marketing data) and insurance claims, together can represent social, medical and behavioral health conditions to form specific relevant subgroup of patients. The proposed patient segmentation framework will facilitate healthcare resource planning and development of interventions to improve the healthcare delivery for each segment. This approach to segmentation will demonstrate heterogeneity in population groups with respect to age, morbidity, lifestyle, setting in which care was mostly used, etc. Therefore, depending on the patterns of utilization of care, complexity level of patients and lifestyle segmentation, various models of care will be needed. For instance, for “young or middle age and healthy” segment that focus little on preventive care and are fans of fast food, the most important approaches may be disease prevention, health education and robust primary care, working with non-healthcare partners such as employers, community-based disease education in order to maintain the health status and promote healthy behavior. Patients with stable but chronic condition that are more interested in adopting technology, can instead benefit more from supportive self-management such as home-based self-monitoring tools to promote health empowerment. Patients with complex chronic conditions that are not managed well and live in neighborhoods with low levels of food access may require more multidisciplinary medical and social care coordination.

Some other examples of the opportunities as a result of linking consumer data to insurance claims and/or EHRs (not limited to patient segmentation) include reduction of obesity through increasing the relevance and effectiveness of weight loss engagement strategies by using consumer lifestyle segmentation variables including diet attitudes and motivations, gauging the receptivity of patients to different outreach channels (automated voice, live agent calls and text messages) using the digital media preference, age and education level, identifying food insecure households/individuals using frequency and dollars spend in food category particularly for individuals living in low-income and low food-access neighborhoods.

Unlike other traditional SDOH data sources that are only available at the county and/or zip code level, such as Area Health Resource Files (38), US Census County Business Patterns (39), and County Health Rankings (40), consumer data is available at the individual or household level. County level social data, although useful, only represent a profile of the community and does not reliably represent the profile of the individual patient. For example, research has shown that poverty is strongly associated with an increase in risk of dying, but simply living in a high-poverty area is not (41).

Despite the important opportunity that the consumer marketing data brings to healthcare, major concerns still exist about privacy of consumers. Linking consumer marketing data to EHRs and/or insurance claims data may increase informational risk (i.e., HIPAA violations), if strict data deidentification standards are not in place and/or data protections are applied inconsistently across various entities which collect, share and use the data (42). As such, any use cases of consumer data must be HIPAA compliant to ensure protection of “individually identifiable health information” (i.e., protected health information) (43). Some of the best practices to ensure compliance are safe sourcing (working with the source compilers of consumer data to ensure compliance), safe storage (reviewing and updating data privacy policies to control access), appropriate/ethical use of data (marketing data should never be used to deny access to anyone or result in health disparities) (44).

Other challenges of using consumer data include reproducibility and analytical challenges. Predictive models developed by the private sector are not shared publicly, therefore cannot be replicated by other researchers to ensure accuracy, validity and potential model bias (34). Additionally, researchers should be cautious when selecting the analytical approaches when it comes to the inclusion of marketing data to predict health outcomes. Highly flexible machine learning algorithms may select features (e.g., reality TV show from consumer interest data) to predict mortality which may not be clinically reasonable.

Despite the challenges discussed above, consumer marketing data may open up opportunities to health researchers to understand how individual level SDOH manifest throughout a person's life. Future patient segmentation models that incorporate SDOH from consumer marketing data have the potential to improve health and reduce health disparities by ensuring that the right patients will be intervened at the right time.

## Author Contributions

MR devised the idea, performed literature review, and wrote the manuscript.

## Conflict of Interest

The author declares that the research was conducted in the absence of any commercial or financial relationships that could be construed as a potential conflict of interest.
